# Comparing Telephone Survey Responses to Best-Corrected Visual Acuity to Estimate the Accuracy of Identifying Vision Loss: Validation Study

**DOI:** 10.2196/44552

**Published:** 2023-03-07

**Authors:** John Wittenborn, Aaron Lee, Elizabeth A Lundeen, Phoebe Lamuda, Jinan Saaddine, Grace L Su, Randy Lu, Aashka Damani, Jonathan S Zawadzki, Colin P Froines, Jolie Z Shen, Timothy-Paul H Kung, Ryan T Yanagihara, Morgan Maring, Melissa M Takahashi, Marian Blazes, David B Rein

**Affiliations:** 1 Public Health Analytics National Opinion Research Center at the University of Chicago Chicago, IL United States; 2 Department of Ophthalmology University of Washington Seattle, WA United States; 3 Centers for Disease Control and Prevention Atlanta, GA United States

**Keywords:** vision, blindness, surveillance, survey, acuity, validation, visual health, optometry clinic, eye disease, vision loss

## Abstract

**Background:**

Self-reported questions on blindness and vision problems are collected in many national surveys. Recently released surveillance estimates on the prevalence of vision loss used self-reported data to predict variation in the prevalence of objectively measured acuity loss among population groups for whom examination data are not available. However, the validity of self-reported measures to predict prevalence and disparities in visual acuity has not been established.

**Objective:**

This study aimed to estimate the diagnostic accuracy of self-reported vision loss measures compared to best-corrected visual acuity (BCVA), inform the design and selection of questions for future data collection, and identify the concordance between self-reported vision and measured acuity at the population level to support ongoing surveillance efforts.

**Methods:**

We calculated accuracy and correlation between self-reported visual function versus BCVA at the individual and population level among patients from the University of Washington ophthalmology or optometry clinics with a prior eye examination, randomly oversampled for visual acuity loss or diagnosed eye diseases. Self-reported visual function was collected via telephone survey. BCVA was determined based on retrospective chart review. Diagnostic accuracy of questions at the person level was measured based on the area under the receiver operator curve (AUC), whereas population-level accuracy was determined based on correlation.

**Results:**

The survey question, “Are you blind or do you have serious difficulty seeing, even when wearing glasses?” had the highest accuracy for identifying patients with blindness (BCVA ≤20/200; AUC=0.797). The highest accuracy for detecting any vision loss (BCVA <20/40) was achieved by responses of “fair,” “poor,” or “very poor” to the question, “At the present time, would you say your eyesight, with glasses or contact lenses if you wear them, is excellent, good, fair, poor, or very poor” (AUC=0.716). At the population level, the relative relationship between prevalence based on survey questions and BCVA remained stable for most demographic groups, with the only exceptions being groups with small sample sizes, and these differences were generally not significant.

**Conclusions:**

Although survey questions are not considered to be sufficiently accurate to be used as a diagnostic test at the individual level, we did find relatively high levels of accuracy for some questions. At the population level, we found that the relative prevalence of the 2 most accurate survey questions were highly correlated with the prevalence of measured visual acuity loss among nearly all demographic groups. The results of this study suggest that self-reported vision questions fielded in national surveys are likely to yield an accurate and stable signal of vision loss across different population groups, although the actual measure of prevalence from these questions is not directly analogous to that of BCVA.

## Introduction

Vision loss and blindness affect approximately 7.08 million Americans and cost the US economy US $134 billion per year [1,2]. Improving and targeting public health and medical interventions to reduce this burden depend on measuring changes in vision loss and blindness prevalence over time at the national, state, and local level. The 2016 National Academies of Sciences, Engineering, and Medicine report *Making Eye Health a Population Health Imperative* [3] highlighted the role that epidemiological surveillance of vision loss could play in mitigating the burden of vision loss. This report included recommendations for the Centers for Disease Control and Prevention (CDC) to develop case definitions to measure visual health, evaluate the utility of self-reported vision measures, and establish a national surveillance system to track vision and eye health indicators in available data sources [3]. A particular challenge to meeting these recommendations is reconciling the different types of data and case definitions used to identify vision loss and blindness prevalence. Historically, most estimates of the prevalence of vision loss and blindness available in the published literature are derived from either population-based examination studies that objectively measure visual function in a defined geographic area and apply these rates to the national population or from nationally representative surveys that measure self-reported vision loss through responses to a wide variety of survey questions [4-8].

Direct measurement of best-corrected visual acuity (BCVA) in the better-seeing eye as evaluated by a trained eye-care technician is accepted as the gold standard method to determine vision impairment or blindness at the person level. Several high-quality population-based studies have attempted to measure BCVA among all persons or persons of a specific race/ethnicity group residing within specific localities [5,6,9-11]. The National Health and Nutrition Examination Survey (NHANES), which approximated BCVA using autorefractors from 1999-2008, is the only examination study derived from a nationally representative sample [4,12]. However, the high cost of examination studies limits their sample size and collection frequency, and study recruitment logistics result in the exclusions of important populations including younger persons, persons with risk factors, the oldest age groups, and persons living in institutional settings such as nursing homes. As a result, examination studies have limited capacity to measure and monitor prevalence for the entire population, identify trends or disparities over time among sociodemographic and risk factor groups, or identify variation in prevalence at the state or local level.

In contrast, survey-based measures of self-reported vision loss and blindness offer advantages of timeliness and much larger sample sizes and typically include richer covariate information on demographic, socioeconomic, behavioral, access to care, and health risk factors. At least 16 nationally representative surveys include self-reported vision questions, including several at the state level and one at the county level [13,14]. Given their low cost of administration, self-reported vision questions can be extremely useful for population health surveillance if they yield a consistent signal of vision problems at the population level, even if they are less precise at the individual level. However, the large number of different survey questions and differences in how they are administered in different surveys result in a wide range of estimates, and experts have expressed skepticism about their accuracy [15,16]. Currently, the validity and correlation of self-reported vision for determining measured visual function is unknown.

Two recent surveillance estimates of the prevalence of vision loss by the CDC’s Vision and Eye Health Surveillance System (VEHSS) and the World Health Organization’s (WHO) Global Burden of Disease project used statistical modeling approaches to combine information from both examination- and survey-based data sources [17]. The VEHSS composite prevalence estimates of blindness and visual acuity loss used Bayesian meta-analytic methods to combine prevalence information from multiple published examination studies that measured BCVA, benchmarked to the NHANES examination measures. The analysis then used self-reported information from the American Community Survey and the National Survey of Children’s Health to predict visual acuity loss prevalence among populations that were excluded or insufficiently captured by examination-based data, including children, older age groups, and persons living in institutional settings, as well as to allocate prevalence by state and county based on the 2019 US population [2]. The WHO estimates similarly combine examination- and survey-based data to estimate burden in WHO regions for which insufficient data exist.

However, more research on the relationship between self-reported vision problems and measured BCVA is vital to better understand the utility of these questions for public health surveillance. Determining the extent to which variation in self-reported vision problems correlates to variation in underlying visual function will support the ongoing development and interpretability of novel surveillance estimates that leverage multiple types of data. In this paper, we sought to estimate the accuracy of self-reported vision loss measures fielded in national surveys by comparing their results to a gold standard of evaluated BCVA at both the individual and population level.

## Methods

We conducted a retrospective chart review and concurrent telephone interview among a sample of patients from the University of Washington (UW) ophthalmology and optometry clinics and calculated statistics to inform the accuracy and validity of telephone survey self-reported visual function compared to examination-measured BCVA. We evaluated findings both at the person level, where we assessed the diagnostic accuracy of the survey questions for identifying BCVA loss, and at the population level, where we compared sample-level prevalence rates derived from survey responses to those based on BCVA.

### Participant Sample

We sampled study participants from current or recent patients from the UW ophthalmology and optometry clinics. First, we restricted a patient-level electronic health record file to only include patients with a visit from May 2018 through April 2020. We then sampled 1200 patients for inclusion such that approximately 20% had diagnoses for age-related macular degeneration, 20% had diagnoses for diabetic retinopathy, and 20% had diagnoses for glaucoma. Among these patients, we attempted to oversample patients with severe or vision-threatening stages of disease. We sampled for 2 additional cohorts of 20% each, which excluded any patients with diagnoses of age-related macular degeneration, diabetic retinopathy, or glaucoma: one cohort in which patients had presenting visual acuity loss of 20/40 or worse in the better-seeing eye and another cohort in which patients had no record of visual acuity loss. The diagnosed prevalence of cataract was so high that we did not need to oversample for patients with this condition. The overall sample size was too low to specifically oversample for additional diseases, so the prevalence of these conditions in the sample was based on the background rate of selected patients. Between the summer of 2020 and the spring of 2021, UW ophthalmology students and residents recruited and obtained informed consent from 669 patients, of whom 438 (65.5%) completed the telephone survey interview.

### Chart Review

We abstracted chart information for all 669 recruited patients from October 2020 until March 2021. The project team developed a secure, web-based form that allowed chart abstractors to complete their reviews remotely (due to COVID-19) and automatically aggregate and store the review information. The form was reviewed by an external panel of experts in ophthalmology, and recommended revisions were incorporated. The form captured presenting (or habitual) acuity and BCVA in each eye and contained additional free-text fields designed to capture patient vision information from electronic health record assessment and treatment plan information. The abstractions were conducted by 2 UW ophthalmology residents (GLS and RL) under the supervision of a professor (AL). Study authors manually reviewed the acuity fields and assigned a single *logMar* value to each patient based on BCVA in the better-seeing eye. These values were re-reviewed by the original abstractors. For reporting purposes, acuity is shown in Snellen acuity ratios.

### Telephone Survey

A telephone survey was administered to consented patients in 2 rounds (November 2020 and May 2021) to minimize the amount of time between the survey and the patients’ recruitment and consent. Nonrespondents from the first round were recontacted in the second round. The survey was administered by professional interviewers with experience conducting federally funded national surveys. The survey included sections with questions related to patient sociodemographic characteristics, eye diseases, visual function, and vision-related difficulties with activities of daily living (ADL). The question order within each section was randomized for each respondent.

To select questions to include in the survey, we first identified vision-related survey questions from 16 recent or ongoing national surveys [14]. We reviewed the questions with the CDC’s Vision Health Initiative and an expert advisory panel convened under the VEHSS, as well as experts in telephone survey administration and psychometrics at NORC at the University of Chicago. We selected three visual function questions from current or recent national surveys: (Question [Q] 1) “Are you blind, or do you have serious difficulty seeing, even when wearing glasses?” which has been administered by the American Community Survey since 2008 and the Behavioral Risk Factor Surveillance System core module since 2013. (Q2) “At the present time, would you say your eyesight, with glasses or contact lenses if you wear them, is excellent, good, fair, poor, or very poor” which is a scaled response question included in NHANES from 1999-2008. (Q3) “Are you blind or unable to see at all?” was administered by the National Health Interview Survey (NHIS) since 1999. We considered but ultimately did not select other questions, including the NHIS question, “Do you have any trouble seeing, even when wearing glasses or contact lenses?” because the wording was deemed to be too similar to Q1.

We also added 2 additional questions not included in prior surveys: “Have you ever been told by a doctor or other health professional that you...” (Q4) “have visual impairment?” and (Q5) “are blind?” which are similar in structure to self-reported eye disease questions that were also included in the survey. We included 6 questions on vision-related ADL that were fielded in the NHIS from 2016-2018, which all began with, “Even when wearing glasses or contacts lenses, because of your eyesight, how difficult is it for you to...” followed by descriptions of ADL, including reading newsprint and driving during the day. Full details and results for the ADL questions are included in Multimedia Appendix 1.

### Analyses

We calculated descriptive statistics for all survey responses and chart abstraction values. To assess diagnostic accuracy of questions at the person level, we calculated the sensitivity, specificity, and area under the receiver operator curve (AUC), comparing survey responses to a gold standard of chart-reviewed BCVA. We did not report positive and negative predictive values because these varied based on the underlying prevalence of vision loss in the sample, and by design, we oversampled for vision loss. For Q1 and Q2, we calculated box-and-whisker plots including the mean, median, and IQR of *logMar* values for each survey response value. To assess the validity of self-reported survey questions for quantifying population-level prevalence rates of BCVA-assessed vision loss and blindness, we compared the sample-level prevalence rate and 95% CI, overall and by subgroup. To test for response bias in the telephone survey, we calculated Kruskal-Wallis or chi-square *P* values for differences in baseline demographic characteristics for respondents compared to nonrespondents (Multimedia Appendix 2).

### Ethics Approval

The study protocol was reviewed and approved by the UW Institutional Review Board (STUDY00008957) and conforms to the Declaration of Helsinki. All patients included in the study provided informed consent for inclusion in the study, including consenting to the telephone interview and secondary analysis of their medical records and claims information. Consent was obtained via telephone due to COVID-19 restrictions on in-person research activities. No person-level data from this study are released. Participants received no compensation for participation in the study.

## Results

### Sample Characteristics

The study sample included 438 patients who completed the telephone survey, which was 65.5% of the 669 patients who consented for participation (Table 1). On average, the interview was conducted 5 months after the most recent acuity measurement. Nearly half (n=215, 49.1%) of the 438 patients were interviewed within 120 days of their last acuity measurement and 86.5% (n=379) were interviewed within a year of their last exam, where the longest time difference was 1 year 3 months. We found no significant differences in AUCs associated with duration of time between the survey and exam. We also found no significant differences in demographic characteristics between patients who did and did not respond to the telephone survey (age: *P*=.53; sex: *P*=.44; and race/ethnicity: *P*=.12; Multimedia Appendix 2). The sample was 54.1% (n=237) female, 70.5% (n=309) non-Hispanic White, 9.6% (n=42) non-Hispanic Black, 4.8% (n=21) Hispanic, and 11.6% (n=51) other races/ethnicities. The majority (n=251, 57.3%) of patients were aged 65-84 years; 25.8% (n=113) were aged 40-64 years, 8% were aged (n=35) 85+ years, 8% were aged (n=35) 18-39 years, and 0.9% were aged (n=4) 0-17 years. Of all the respondents, 38.4% (n=168) reported education beyond a bachelor’s degree, 28.8% (n=126) had an associate’s or bachelor’s degree, and 32.4% (n=142) had less than a college degree. Nearly two-thirds (n=281, 64.2%) of patients had BCVA better than 20/40, equating to normal vision; 17.8% (n=78) had mild impairment of 20/40 to <20/80; 4.8% (n=21) had moderate impairment of 20/80 to <20/200; and 13.2% (n=58) were blind, including 2.1% (n=9) with 20/200 to <20/400 and 11.2% (n=49) with blindness defined as acuity ≤20/400, difficulty counting fingers or seeing hand motions, or having no light perception.

**Table 1:** Demographic characteristics of the study population.

Characteristic		Patient (n=438), n (%)
**Sex**		
	Male	199 (45.4)
	Female	237 (54.1)
**Age (years)**		
	0-17	4 (0.9)
	18-39	35 (8)
	40-64	113 (25.8)
	65-84	251 (57.3)
	85+	35 (8)
**Race/ethnicity**		
	Hispanic	21 (4.8)
	Non-Hispanic Black	42 (9.6)
	Non-Hispanic White	309 (70.5)
	Other	51 (11.6)
	Unknown	15 (3.4)
**Education**		
	Less than college degree	142 (32.4)
	Associate’s or bachelor’s degree	126 (28.8)
	Graduate degree	168 (38.4)
	Unknown	2 (0.5)
**Best-corrected visual acuity in the better-seeing eye**		
	>20/40 (normal vision)	281 (64.2)
	20/40 to <20/80 (mild vision impairment)	78 (17.8)
	20/80 to <20/200 (moderate vision impairment)	21 (4.8)
	20/200 to <20/400 (blindness)	9 (2.1)
	≤20/400 (blindness based on acuity, CF^a^, HM^b^, or NLP^c^)	49 (11.2)

^a^CF: counting fingers.

^b^HM: hand motion.

^c^NLP: no light perception.

### Survey Responses

**Figure 1:**
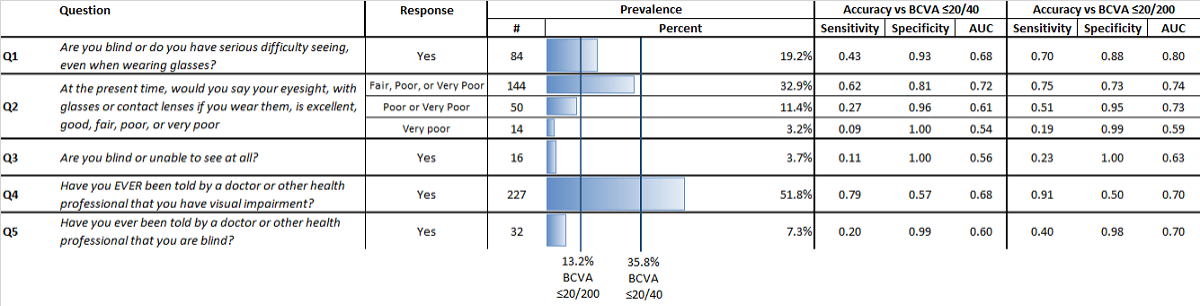
Sample prevalence, sensitivity, specificity, and area under the receiver operator curve (AUC) of survey questions versus any vision loss or blindness. The sample prevalence of survey response options compared to the gold standard of best-corrected visual acuity (BCVA) thresholds of ≤20/40 (vision loss) and ≤20/200 (blindness) is shown. The diagnostic accuracy of each question is compared to BCVA-assessed vision loss or blindness. Sensitivity (sens.) refers to the rate at which the question accurately identifies vision loss or blindness in comparison to BCVA (true positive), whereas specificity (spec.) refers to the rate at which the question correctly identified BCVA vision, indicating no vision loss or blindness (true negative). AUC measures both sensitivity and specificity and, therefore, is the primary measure of diagnostic accuracy reported in this analysis. Q: question.

Of respondents, 19.2% (n=84) self-reported “yes” to the question Q1, “Are you blind or do you have serious difficulty seeing, even when wearing glasses?”; 3.7% (n=16) responded that they are “blind or unable to see at all” (Q3); and 55.5% (n=227) and 7.3% (n=32) of respondents stated they had ever been told by a doctor that they had visual impairment (Q4) and blindness (Q5), respectively (Figure 1).

### Person-Level Accuracy of Survey Responses for Indicating BCVA

**Figure 2:**
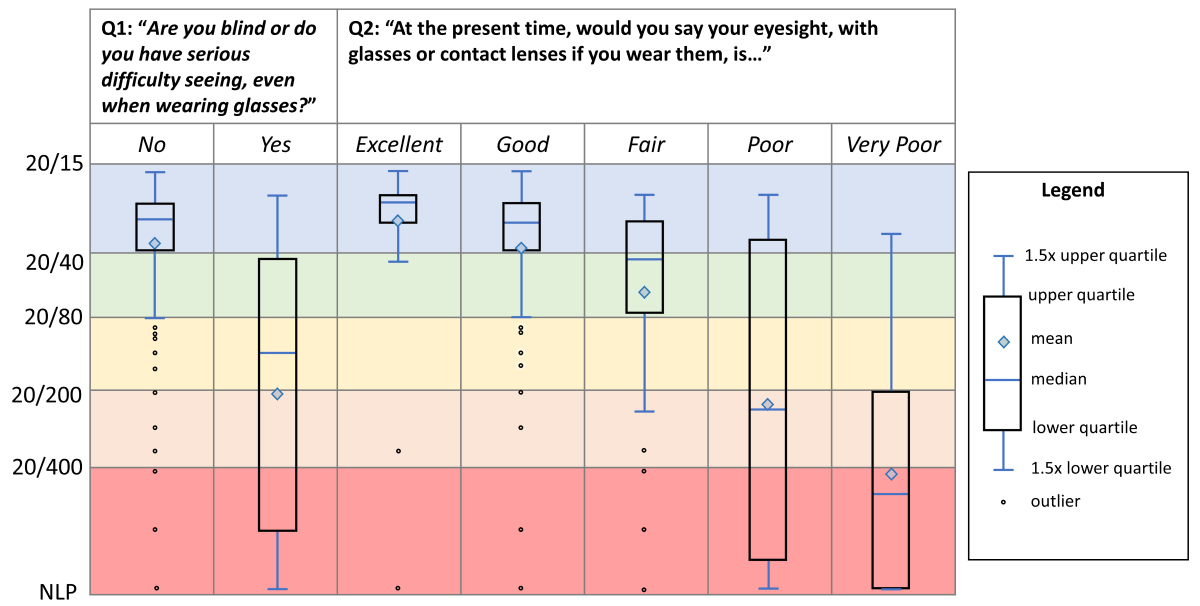
Distribution of best-corrected visual acuity (BCVA) in the better-seeing eye by survey response value. The box-and-whisker plots show the distribution of BCVA among patients who gave a response to both Q1 and Q2. These questions were selected because they were the 2 questions with the highest diagnostic accuracy for BCVA blindness and vision loss, respectively. The box-and-whisker plots show the mean, median, upper and lower quartiles, 1.5× quartiles, and outliers defined as values outside of the range of 1.5× the lower and upper quartiles. The distribution of BCVA generally corresponds with the survey response. However, for Q2, “Excellent” and “Good” responses are both primarily within the normal vision range, whereas “Poor” responses exhibits a very wide distribution. NLP: no light perception; Q: question.

Figures 1 and 2 show associations between BCVA and survey responses at the person level. Figure 1 reports the sensitivity, specificity, and AUC of each survey question for predicting either vision loss (≤20/40) or blindness (≤20/200). For any vision loss, Q4 had the highest sensitivity (0.79), whereas Q3 had 100% specificity. Q2 achieved the highest AUC (0.72) and the smallest percentage difference when including any response of “fair,” “poor,” or “very poor” to indicate vision loss (data not shown). Q1 had the second highest AUC of 0.68, but this appears to be achieved through high specificity (0.930) despite relatively low sensitivity (0.43). Q1 also underestimated vision loss by 46%. For blindness, the highest sensitivity and specificity were achieved by Q4 (0.91) and Q3 (0.995), respectively. Q1 had the highest AUC at 0.80 but overestimated blindness by 47% relative to BCVA. The second highest AUC was achieved by Q2 (0.74) when including responses of “fair,” “poor,” or “very poor” to indicate blindness. Q2 responses of “poor” or “very poor” resulted in the closest overall prevalence estimate for blindness, underpredicting blindness by 12% relative to BCVA. Multimedia Appendix 3 shows the receiver operating characteristic curves associated with these results.

Figure 2 includes box plots depicting the distribution of visual acuity values by survey responses to Q1 and Q2 (selected because these questions achieved the highest AUC values for blindness and vision loss, respectively). For responses to Q1*,* the IQR of “no” responses is almost entirely within the range of normal acuity values of <20/40, whereas the lower threshold of 1.5 × IQR for “no” is almost entirely within the range of mild visual impairment of 20/40 to < 20/80. The IQR of “yes” responses ranges from just over 20/40 to <20/400, but the upper threshold of 1.5 × IQR is nearly 20/20. For Q2, the box plots show that the mean and median acuity values of the 5-response options fall within 5 corresponding levels of acuity loss, although the IQR and outer thresholds span multiple acuity categories.

### Population-Level Vision Loss and Blindness Prevalence

**Figure 3:**
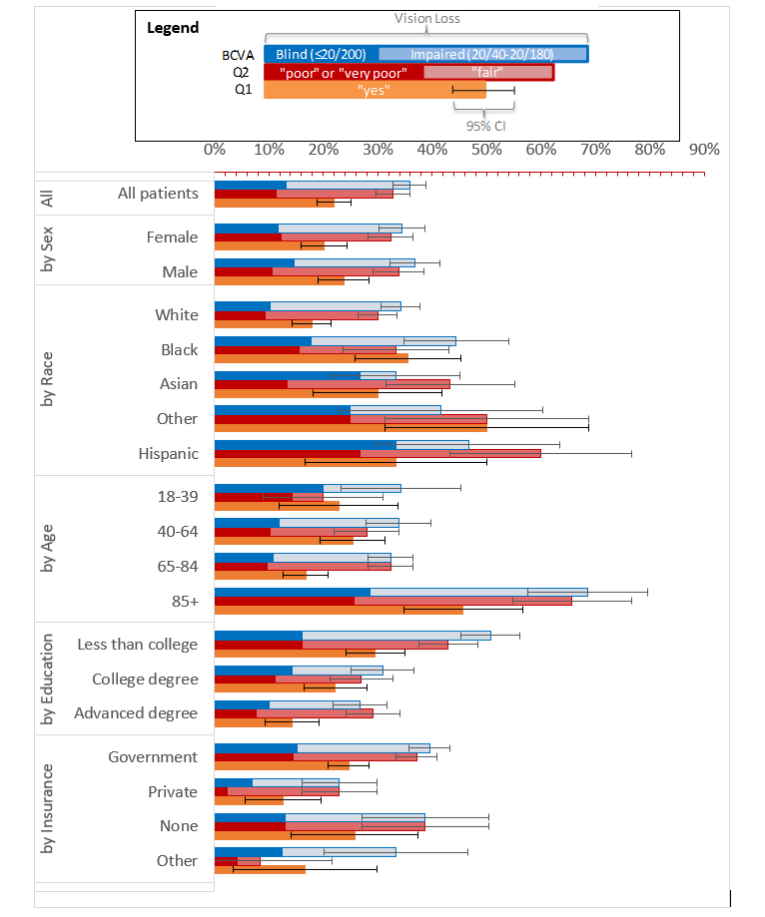
Sample-level prevalence rates of best-corrected visual acuity (BCVA)–assessed blindness and visual impairment, Q2 responses of “poor" or "very poor” and “fair,” and Q1 response of “yes.” The relationship between population-level prevalence rates based on BCVA, Q1, and Q2 at the population level is shown. BCVA is based on the better-seeing eye. Q1 is “Are you blind or do you have serious difficulty seeing, even when wearing glasses?” and Q2 is ”At the present time, would you say your eyesight, with glasses or contact lenses if you wear them, is excellent, good, fair, poor, or very poor?” For each population group, there are 3 bars. The top blue bar is BCVA and depicts 2 stacked bars. The darker blue bar on the left is the prevalence of blindness (≤20/200). The lighter blue bar stacked to the right is the prevalence of persons with visual impairment (20/40 to 20/180). Thus, the sum of the upper, blue stacked bars represents any vision loss (≤20/40). The red stacked bar below depicts prevalence results from Q2. The dark red bar on the left includes patients who reported “poor” or “very poor” eyesight. The lighter red bar stacked to the right is the prevalence of “fair” responses. Thus, the value of the dark and light red stacked bars represents patients who reported “very poor,” “poor,” or “fair” eyesight. The lower orange bar is the prevalence of “yes” responses to Q1. Q: question.

Figure 3 shows the population-level prevalence rates for BCVA and Q1 and Q2 responses overall and by sex, race/ethnicity, age group, insurer, and education status among the sample. BCVA is shown as a stacked bar with blindness on the left and impairment on the right; the cumulative sum of these bar charts is equivalent to any BCVA loss. The prevalence of vision loss for all patients was 35.8% (157/438). Responses for Q2 are similarly stacked, such that the left bar includes responses of “poor” or “very poor” and the right bar includes “fair”; thus, the stacked cumulative total would equate to the responses of “fair,” “poor,” or “very poor,” which had an overall prevalence of 32.9% (n=144). Q1 “yes” responses had an overall prevalence of 21.9% (96/438).

At the population level, Q1 and Q2 responses exhibit similar trends and disparities in prevalence rates as those of BCVA. The two Q2 response groupings are closely correlated with the 2 BCVA categories. For Q2, the relative difference in prevalence rate of “poor” or “very poor” responses was 14% lower than that of BCVA blindness, whereas the prevalence of Q2 responses of “fair,” “poor,” or “very poor” was 8% lower than BCVA vision loss. Q1 response of “yes” relatively overpredicts BCVA blindness (≤20/200) by 66% and underpredicts BCVA vision loss (≤20/40) by 39%. The relative relationships in prevalence between BCVA, Q1, and Q2 were stable across most sample subgroups. Prevalence based on Q1 falls within the range of BCVA blindness to vision loss for every subgroup except the “Other race” subgroup, although this result is based on a sample of only 12 patients. Similarly, the cumulative responses from Q2 fell within the range of blindness to vision loss for all subgroups except the “Other race,” “Hispanic,” “Advanced degree,” and “Other insurance” subgroups.

## Discussion

This study provides evidence on the accuracy and validity of vision and eye health survey questions, including those currently or recently deployed through federally funded national surveys. We measured the concordance between self-reported vision loss or blindness and vision-related ADL limitations versus vision measured with BCVA among a sample of patients from ophthalmology and optometry clinics and reported results both at the person and population level. The highest AUC for predicting blindness (0.80) was achieved by Q1, “Are you blind, or do you have serious difficulty seeing, even when wearing glasses?” The highest AUC (0.72) for predicting any BCVA loss was achieved by Q2, “At the present time, would you say your eyesight, with glasses or contact lenses if you wear them, is…” when the responses of “fair,” “poor,” or “very poor” are used to indicate any vision loss. Prevalence rates based on Q2 or adjusted Q1 responses were highly correlated to population-level prevalence of BCVA blindness and vision loss and suggest that at the population level, variation in self-reported prevalence based on Q1 and Q2 is likely to accurately reflect patterns and variations in the underlying prevalence of visual acuity loss and blindness.

This study has several limitations. Our study sample consisted of current or recent patients from optometry and ophthalmology clinics, which are not representative of the general population and may have higher awareness of their visual health. This sample also exhibited higher levels of education and, due to our intentional oversampling, a much higher prevalence of evaluated BCVA impairment than would be seen in a nationally representative population. Due to COVID-19–related restrictions on in-person research, patient acuity measures were collected through retrospective chart review. Among the 669 recruited patients, 231 (34.5%) did not complete a telephone interview. In addition, we define vision loss based on BCVA only. We did not consider other measures of vision loss such as reduced visual field, contrast sensitivity, or near-distance acuity because this information was not routinely recorded in the medical charts. Due to this limitation, we likely misclassified some patients with better vision. We additionally compared self-reported values to BCVA as opposed to presenting visual acuity because of data quality concerns regarding how consistently presenting or habitual acuity may have been recorded in patient charts and because historically, vision loss and blindness prevalence estimates are reported based on BCVA. Finally, time differences between the patients’ survey interview and the date of their nearest examination may bias results. Presumably, longer time periods would reduce the apparent accuracy of the self-reported responses, although we did not find a significant association between this time difference and AUC.

Identifying and documenting the concordance and relationship between self-reported vision problems and BCVA is important to enhance national surveillance of vision loss and eye problems. Historically, visual health epidemiological estimates were derived from either national surveys or examination studies, each of which have inherent strengths and limitations. Examination studies provide estimates of objectively measured vision loss, but due to their high cost and complexity, they are all based on small, localized samples, many of which are arguably out of date. National self-reported surveys provide large, representative, and ongoing samples that examination studies lack, but self-reported instruments previously lacked evidence of validity as proxy measures of actual vision loss.

The purpose of this study was to evaluate the validity of self-reported vision measures to assess population health. The results of this study imply that self-reported questions should not be used as a substitute for clinical vision evaluation but do support the interpretation of self-reported survey questions as proxy measures of variation in population visual health for use in surveillance. The recent surveillance estimates of the prevalence of visual acuity loss produced by the CDC’s VEHSS and the WHO both leverage the strengths of examination studies (their robust measurement of vision loss) as well as that of national surveys (their large, ongoing representative samples) to provide more complete and detailed epidemiological estimates than can be supported by either type of data alone [2,17]. Our findings lend support to the methodologies and assumptions of these ongoing national and international visual health surveillance efforts by measuring the accuracy of self-reported vision at the individual and population level.

## Appendix

Multimedia Appendix 1 Activities of daily living (ADL) questions.

Multimedia Appendix 2 Baseline characteristics for all consented patients.

Multimedia Appendix 3 Receiver operator curves (ROC).

## Abbreviations

ADL: activities of daily living
AUC: area under the receiver operator curve
BCVA: best-corrected visual acuity
CDC: Centers for Disease Control and Prevention
NHANES: The National Health and Nutrition Examination Survey
NHIS: National Health Interview Survey
Q: question
UW: University of Washington
VEHSS: Vision and Eye Health Surveillance System
WHO: World Health Organization

## Data Availability

Data collected for this study are restricted per the institution review board approval at the University of Washington and are not publicly available.
