# Supplementary material for: Comparing Telephone Survey Responses to Best-Corrected Visual Acuity to Estimate the Accuracy of Identifying Vision Loss: Validation Study
Source: JMIR Public Health Surveill. 2023 Mar 7;9:e44552. doi: 10.2196/44552 (PMC10031446; doi:10.2196/44552)
Supplement: Multimedia Appendix 1 [file publichealth_v9i1e44552_app1.docx]

**Multimedia Appendix 1.** **Activities of Daily Living (ADL) Questions.**

In addition to survey questions intended to indicate the presence of vision loss, we also included questions related to activities of daily living (ADL) which may indicate the impact of vision loss. We included six questions on vision related ADLs that were fielded in the NHIS which all began with “Even when wearing glasses or contacts lenses, because of your eyesight, how difficult is it for you to…” followed by activity descriptions, including reading newsprint (Q7), up-close work or hobbies (Q8), going down steps in dim light (Q9), noticing objects to the side (Q10), finding something on a crowded shelf (Q11), and driving during the daytime (Q12). These questions are similar to ADL questions that were also previously fielded in NHANES.

**Table S1** shows the distribution of responses to each ADL question. Each row sums to 100%, with darker shaded cells indicating more frequent responses.

**Table S1. Frequency of Responses to Questions on Limitations in Activities of Daily Living Due to Eyesight.**

**Table S2** shows the distributions of scaled survey question responses by ranges of best corrected visual acuity (BCVA) loss. Each row, representing a BCVA range, sums to 100%, with darker shading representing a higher proportion of respondents within that BCVA who reported each survey response option. The six functional limitation questions are shown by 3 acuity ranges. For example, among persons with normal BCVA, 59% said that reading ordinary newsprint is not at all difficult. All questions show positive correlation between acuity loss and self-reported severity scale. The highest Pearson correlation was achieved by Q10 (objects to side) at 45.5%, followed by Q7 (newsprint) at 43.4%, Q12 (driving) at 39.7%, Q11 (crowded shelf) at 39.4%, Q9 (steps at night) at 37.5% and Q8 (work or hobbies up close) at 35.9%.

**Table S2. Distribution of Responses to Six Questions on the Impact of Eyesight on Activities of Daily Living (ADLs).**

| ***"Even when wearing glasses or contacts lenses, because of your eyesight, how difficult is it for you to":*** | | | | | |
| --- | --- | --- | --- | --- | --- |
|  | *"Not at all difficult"* | *"Only a little difficult"* | *"Somewhat difficult"* | *"Very difficult"* | *"Can't do at all because of eyesight"* |
| *"read ordinary print in newspapers?"* |  |  |  |  |  |
| 1-Normal (20/15-20/30) | 59% | 19% | 19% | 3% | 0% |
| 2-Impairment (20/40-20/150) | 25% | 27% | 30% | 13% | 6% |
| 3-Blind (20/200+) | 26% | 12% | 16% | 22% | 24% |
| *"do work or hobbies that require you to see well up close "* | |  |  |  |  |
| 1-Normal (20/15-20/30) | 61% | 20% | 16% | 3% | 0% |
| 2-Impairment (20/40-20/150) | 27% | 27% | 31% | 13% | 2% |
| 3-Blind (20/200+) | 28% | 17% | 19% | 28% | 9% |
| *"go down steps, stairs, or curbs in dim light or at night"* | |  |  |  |  |
| 1-Normal (20/15-20/30) | 53% | 28% | 15% | 4% | 1% |
| 2-Impairment (20/40-20/150) | 29% | 26% | 29% | 16% | 0% |
| 3-Blind (20/200+) | 22% | 18% | 25% | 29% | 5% |
| *"notice objects off to the side while you are walking"* | |  |  |  |  |
| 1-Normal (20/15-20/30) | 73% | 17% | 9% | 2% | 0% |
| 2-Impairment (20/40-20/150) | 48% | 25% | 22% | 3% | 1% |
| 3-Blind (20/200+) | 17% | 29% | 21% | 24% | 9% |
| *"find something on a crowded shelf"* |  |  |  |  |  |
| 1-Normal (20/15-20/30) | 78% | 13% | 7% | 3% | 0% |
| 2-Impairment (20/40-20/150) | 53% | 23% | 19% | 3% | 1% |
| 3-Blind (20/200+) | 39% | 12% | 23% | 25% | 2% |
| *"drive during the daytime in familiar places"* |  |  |  |  |  |
| 1-Normal (20/15-20/30) | 85% | 9% | 3% | 1% | 2% |
| 2-Impairment (20/40-20/150) | 64% | 9% | 16% | 1% | 10% |
| 3-Blind (20/200+) | 32% | 8% | 2% | 2% | 57% |
|  |  |  |  |  |  |
| Shading Scale: | 0% | 25% | 50% | 75% | 100% |
