# Supplementary material for: Comparing Telephone Survey Responses to Best-Corrected Visual Acuity to Estimate the Accuracy of Identifying Vision Loss: Validation Study
Source: JMIR Public Health Surveill. 2023 Mar 7;9:e44552. doi: 10.2196/44552 (PMC10031446; doi:10.2196/44552)
Supplement: Multimedia Appendix 2 [file publichealth_v9i1e44552_app2.docx]

**Multimedia Appendix 2. Baseline Characteristics for All Consented Patients**

|  | **Completed Telephone Survey?** | | **Total (N=669)** | ***P-value*** |
| --- | --- | --- | --- | --- |
|  | **No (N=231)** | **Yes (N=438)** |  |  |
|  |  |  |  |  |
| **Age** |  |  |  | 0.53 ^1^ |
| N | 231 | 438 | 669 |  |
| Mean (SD) | 65.3 (16.82) | 66.5 (15.30) | 66.1 (15.84) |  |
| Median | 69.0 | 69.0 | 69.0 |  |
| Range | 16.0, 98.0 | 18.0, 99.0 | 16.0, 99.0 |  |
|  |  |  |  |  |
| **Sex**, n (%) |  |  |  | 0.44 ^2^ |
| #N/A | 0 (0.0%) | 2 (0.5%) | 2 (0.3%) |  |
| Female | 119 (51.5%) | 238 (54.3%) | 357 (53.4%) |  |
| Male | 112 (48.5%) | 198 (45.2%) | 310 (46.3%) |  |
|  |  |  |  |  |
| **Race/Ethnicity**, n (%) |  |  |  | 0.12 ^2^ |
| AIAN | 4 (1.7%) | 6 (1.4%) | 10 (1.5%) |  |
| Asian | 25 (10.8%) | 30 (6.8%) | 55 (8.2%) |  |
| Black, non-Hispanic | 22 (9.5%) | 45 (10.3%) | 67 (10.0%) |  |
| Hispanic | 13 (5.6%) | 15 (3.4%) | 28 (4.2%) |  |
| Unknown / Other | 12 (5.2%) | 12 (2.7%) | 24 (3.6%) |  |
| White, non-Hispanic | 155 (67.1%) | 330 (75.3%) | 485 (72.5%) |  |
|  |  |  |  |  |
| **Insurance**, n (%) |  |  |  | 0.49 ^2^ |
| Medicaid | 23 (10.0%) | 38 (8.7%) | 61 (9.1%) |  |
| Medicare | 130 (56.3%) | 258 (58.9%) | 388 (58.0%) |  |
| No Insurance | 19 (8.2%) | 31 (7.1%) | 50 (7.5%) |  |
| Other insurance | 6 (2.6%) | 23 (5.3%) | 29 (4.3%) |  |
| Private | 53 (22.9%) | 87 (19.9%) | 140 (20.9%) |  |
| Unknown Insurance | 0 (0.0%) | 1 (0.2%) | 1 (0.1%) |  |
| ^1^ Kruskal-Wallis *P*-*value*; ^2^ Chi-Square *P*-*value* | | | | |

Abbreviations: American Indian / Alaska Native (AIAN).

*Education is not included because this was collected as part of the survey; educational status for patients who did not respond to the survey is unknown.
