# Supplementary material for: Comparing Telephone Survey Responses to Best-Corrected Visual Acuity to Estimate the Accuracy of Identifying Vision Loss: Validation Study
Source: JMIR Public Health Surveill. 2023 Mar 7;9:e44552. doi: 10.2196/44552 (PMC10031446; doi:10.2196/44552)
Supplement: Multimedia Appendix 3 [file publichealth_v9i1e44552_app3.docx]

**Multimedia Appendix 3. Receiver Operator Curves (ROC) of Self-report Survey Responses**


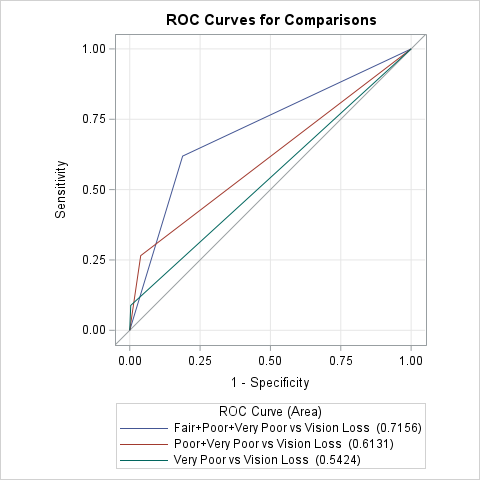

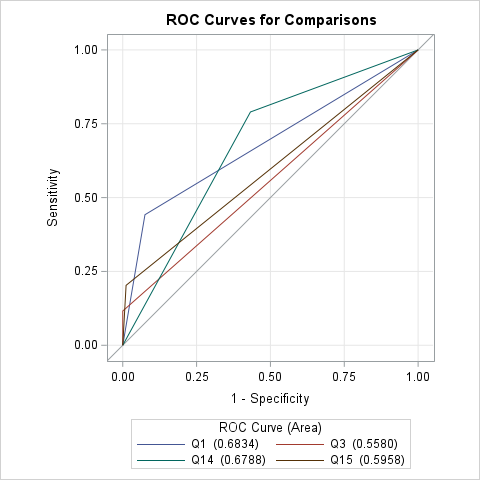
**versus Any Vision Loss** **(≤20/40, best corrected visual acuity):**


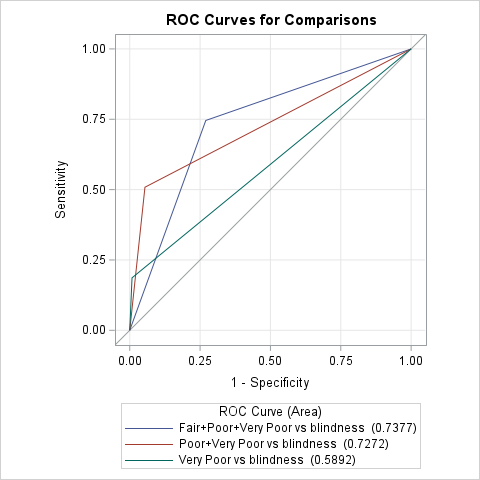

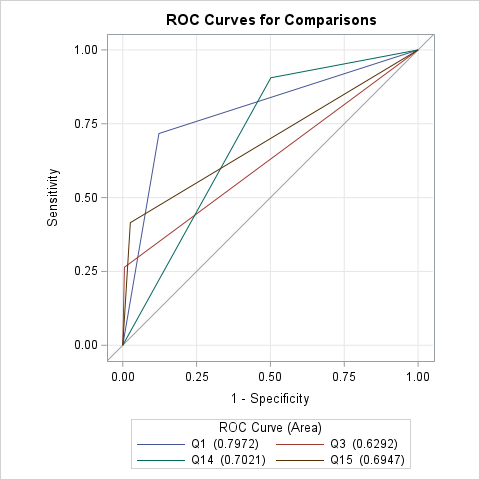
**versus Blindness (≤20/200, best corrected visual acuity):**

**Left figures:**

- Q1= “Are you blind or do you have serious difficulty seeing, even when wearing glasses?”
- Q3=”Are you blind or unable to see at all?”
- Q14=”Have you ever been told by a doctor or other health profession that you have visual impairment?”
- Q15=”Have you ever been told by a doctor or other health profession that you are blind?”

**Right Figures:**

”At the present time, would you say your eyesight, with glasses or contact lenses if you wear them, is excellent, good, fair, poor, or very poor?”
